# Annotations

**Published:** 1892-10-29

**Authors:** 


### Annotations.

**The Methods and Morals of Somerset House.**

A "responsible correspondent" of the *Daily Chronicle* demands a "thorough exposure of Somerset House" and its chemical analysts. We are strongly inclined to the opinion that this correspondent knows what he is about. "In one case," says the *Chronicle*, "a public analyst found fifteen per cent. of foreign fat in a sample of butter. The President of the Society of Analysts confirmed the analysis. But the Somerset House chemists reported that, although the butter was of the 'poorest quality of genuine butter,' there was no evidence of the presence of foreign fat. To which the analysts reply that butter-fat is butter-fat, and that if it is 'poor,' that is because foreign fat has been added." There is a discrepancy here which reflects the gravest possible discredit upon either the President of the Society of Analysts or the analysts of Somerset House. We think it highly probable that the discredit does not attach to the President of the Society of Analysts. There seems to be some ground for believing, first, that the Somerset House analysts are not exceedingly competent persons; and secondly, that they view adulterations and adulterators with a very lenient eye. Now, we submit that it is no part of the business of the Somerset House analysts to be either lenient or severe. Their one and only business is to be expert and exact analytical chemists, and to secure the confidence of the public in that capacity. They have for a long time assumed a very lofty attitude, both of competence and of public morals; and, unfortunately, the public has taken them at their word. One result is pretty obvious, and that is, that adulterators are, in a quiet way, having an uncommonly good time of it. The mere suspicion of such a state of things is an intolerable scandal, and the analysts of Somerset House must take prompt steps to resuscitate their falling reputation.

**The "Practical Man."**

Dr. H. Rainsford, whose letter we reprint elsewhere, says: "I have read with pleasure your leader on 'The Practical Man.' *Apropos* of that I once had a dispenser, a third year's student of St. Mary's, who informed me that in his opinion a senior student was a much better doctor than the ordinary general practitioner." Now that poor young fellow was evidently a very witless goose indeed, and Dr. Rainsford no doubt gave him what he needed most, his indulgent pity. But what shall we say of the physicians and surgeons of St. Mary's, under whose teaching such a hopelessly foolish state of mind had been engendered? Of course, there will always be among young men, here and there one, whose conceit will be so uncontrollable that he will think himself much cleverer than all the rest of the world, his teachers included. This cannot be helped; it is, as Carlyle insists, the nature of the youthful male animal. But the really serious part of the business is that there is a prevailing impression right through the medical student world, that the average general practitioner is an inferior person. The same impression prevails among hospital nurses as amongst medical students, and to the same universal extent. How has it arisen? How has it become an established hospital tradition? It can only have arisen from the constant teaching and influence of those who make and guide public opinion in the little world of the Medical School. The teachers, and the teachers only, are responsible. That the tradition is an unjust and absurd one goes without saying. If one could possibly believe that practical experience teaches the doctor nothing, but makes him actually less capable than he was as an inexperienced student, then we must believe that doctors, as a class, are made of totally different mental stuff from every other class of human beings in the world. For all the world admits that experience is of prime value in every other walk of life than medicine. So much is this the case that a leading daily newspaper the other morning denounced the proposal of the new Chicago University to start a course of lectures and professional instruction for journalists, on the sole ground that nothing but actual experience in journalism can make a man a journalist. General practitioners ought to be encouraged in every way to rely upon themselves, and the public should be encouraged to trust them. Happily, the average doctor is a man of sense and culture, and refuses to be made a mere jackal for the youthful hospital lion.

**Why is "The Hospital" "The Hospital?"**

The answer to the question why is The Hospital The Hospital? is that The Hospital is The Hospital because it is, and because it cannot be anything else. This is a simple way of replying to the criticism contained in Dr. Rainsford's letter. "Perhaps," says Dr. Rainsford, "you will pardon my saying that I cannot see why there should be any articles on *treatment* in a weekly intended for *nurses*." We will not only pardon our correspondent, but thank him for giving us an opportunity of explaining the position of our journal. In the first place, The Hospital is essentially The Hospital it is not The Nurse: that is to say, it aims at reproducing in print, not merely the nursing department, but the whole life and activity of hospitals. Now the life and activity of hospitals includes three notions and facts (1) medical practice, (2) nursing, (3) philanthropy. If, therefore, such a paper as The Hospital is to be published, on what ground of reason can we so much as call it The Hospital, if it is to leave out the one element of activity which alone is fundamental and essential to the idea of a hospital, namely, medical and surgical practice. The Hospital is a medical journal first, just as medicine is the first, and, indeed, the only absolutely indispensable thing in concrete hospital life; it is a nursing journal, secondly, because nursing is now an indispensable adjunct—to what? To the hospital idea? No: but to medical and surgical practice! It is a philanthropic journal in the third place, because in this country a hospital system, the noblest hospital system in the world, has been established on the basis of a great and glorious philanthropy. If it be said that "nurses buy and read our paper, but that in reading it they can read about treatment," surely the answer is obvious! Do not the nurses do their work in the hospital wards? and do they not there both see the treatment and hear all about it from the lips of the physicians and surgeons? If a nurse wishes to learn treatment, she can learn as much in a week by what she sees, and cannot help seeing in her daily work, as she learns in a month or a year by mere reading. Besides, do not the *Lancet* and the *British Medical Journal* deal with treatment? And do not the nurses read them? One other claim we think we may fairly make, and that is, we are loyal to the medical profession; loyal in heart and soul. Hospitals owe everything, their existence in their present form, their noble deeds, their world-wide fame, to medicine and to medical men. The Hospital, if it has any just conception of the nature and range of its high duties, is bound to be consistently loyal to medicine and to its self-denying professors. We will defy our bitterest enemies—if we have any enemies—to say that we have ever exalted nursing at the expense of medicine. Nursing is medicine's handmaid, and can never be anything else.

